# Association between Single Nucleotide Polymorphisms in Monoamine Oxidase and the Severity of Addiction to Betel Quid

**DOI:** 10.3390/cimb46020064

**Published:** 2024-01-23

**Authors:** Chung-Chieh Hung, Ying-Chin Ko, Chia-Min Chung

**Affiliations:** 1 School of Medicine, Chung Shan Medical University, Taichung 40201, Taiwan; cshy2135@csh.org.tw; 2 Department of Psychiatry, Chung Shan Medical University Hospital, Taichung 40201, Taiwan; 3 Department of Medical Research, China Medical University Hospital, China Medical University, Taichung 40447, Taiwan; ycko0406@gmail.com; 4 Graduate Institute of Toxicology, College of Medicine, National Taiwan University, Taipei 106216, Taiwan; 5 Graduate Institute of Biomedical Sciences, China Medical University, Taichung 40402, Taiwan; 6 Department of Psychiatry and Center for Addiction and Mental Health, China Medical University Hospital, Taichung 404327, Taiwan

**Keywords:** betel quid addiction, genetic polymorphism, monoamine oxidase

## Abstract

Betel quid (BQ) is the fourth most popular psychoactive substance in the world, and BQ use disorder (BUD) is prevalent in Asian countries. Although the mechanisms underlying BUD remain unclear, studies have reported influences from monoamine oxidase inhibitor. We enrolled 50 patients with BUD and assessed their BQ consumption habits, emotional conditions, and the clinical severity of addiction—assessed using the *Diagnostic and Statistical Manual of Mental Disorders* [Fifth Edition] (*DSM-5*) criteria, Substance Use Severity Rating Scale, and Yale–Brown Obsessive Compulsive Disorder Rating Scale for BQ. Patients were categorized into the severe group when showing six or more symptoms defined by *DSM-5*. A genome-wide association study was conducted for single nucleotide polymorphisms in *BRCA1*, *COL9A1*, *NOTCH1*, *HSPA13*, *FAT1*, and *MAOA* by using patients’ blood samples. More severe BUD symptoms were associated with younger age of using BQ and poor oral hygiene and with severe craving for and more anxiety toward BQ use. The *MAOA* rs5953210 polymorphism was significantly associated with severe BUD (odds ratio, 6.43; 95% confidence interval, 5.12–7.74; *p* < 0.01) and might contribute to BQ-associated cancer risk. Further studies are required to investigate the addictive properties of BQ and the development of novel diagnostic tools and pharmacotherapeutic alternatives to BUD treatment.

## 1. Introduction

Approximately 600 million people consume betel quid (BQ) worldwide, rendering it the fourth most popular psychoactive substance [1]. BQ use disorder (BUD) has been reported to be a prevalent addictive disorder in Asian countries [2]. In addition, oral and pharyngeal cancers are some of the most prevalent cancers in the world, and in Taiwan, oral cancers are among the 10 most common cancers among men [3]. Studies have demonstrated that BQ consumption is an independent risk factor for oropharyngeal cancers, potentially contributing to carcinogenesis [4,5]. Although the mechanism underlying BQ becoming an addictive substance remains unclear, studies have reported on its influences and interactions with monoamine oxidase inhibitors (MAOIs) [6]. A report suggested that arecoline has monoamine oxidase-A (MAO-A) inhibitor-like properties [7]. The MAO-A inhibitors prevent the neurotransmitter breakdown, thus increasing dopamine and serotonin concentrations in the brain. A reduction of daily BQ use was observed among patients with depression after antidepressant therapy, including MAO-A inhibitors and selective serotonin reuptake inhibitors (SSRIs) [8]. Other investigations have revealed that areca alkaloids act as competitive inhibitors of γ-aminobutyric acid receptors [9], partial agonists of nicotinic acetylcholine receptors [10], and potential activators of dopaminergic neurons [11].

A retrospective study indirectly revealed that antidepressants could reduce the consumption and addictive severity of BQ in patients with depression [12]. In addition, a clinical trial investigating the effect of antidepressants on BQ chewing cessation therapy revealed that antidepressants could effectively reduce BQ consumption among patients with BUD in 2–4 weeks [13]. However, BUD can only be diagnosed through clinical interviews and by using psychometric rating scales, leading to possible bias. Therefore, developing novel diagnostic tools based on reliable biomarkers for evaluating the addiction severity of BQ is crucial; identifying such biomarkers would also help with confirming a diagnosis of BUD.

Investigations regarding the association of single nucleotide polymorphisms (SNPs) and BUD would lead to a better understanding of the addictive property of BQ; such studies could also help identify populations with vulnerabilities to BQ addiction. Most studies on the topic have mainly focused on the carcinogenic effects of BQ. For example, research identified *BRCA1*-associated protein 1 (BAP1) as a tumor suppressor that is possibly related to the development of salivary duct and intracapsular carcinoma [14]. *BRCA1* was also reported to be related to many kinds of cancers [15]. Furthermore, one study assessed a risk model involving the expression of *COL9A1* and *FAT1* in oral malignancies independent of BQ dependence [16]. In addition, studies of genetic interactions have implicated a role of Notch pathway molecules as independent indicators of oral carcinoma [17,18]. In another study, *NOTCH1*, *BRCA1*, *COL9A1*, and *HSPA13* were revealed to be related to the risk of BQ-related development of oral squamous cell carcinoma [19,20]; however, their precise association with the addictive property of BQ remains unclear. Monoamine oxidase A (MAOA) was reported to be related to alcohol and tobacco addiction [21]. However, no study has investigated the association between MAOA and BQ addiction. Research revealed that heavy users of BQ may harbor MAOA gene variants [6]. Because these studies have mainly focused on the carcinogenic effects of BQ, the abovementioned genes that have been implicated in the risk of BQ-related carcinogenesis may be responsible for its addictive properties.

In this study, we investigated the characteristics of patients with BUD with three levels of addiction severity by using clinical interviews, psychometric questionnaires, and emotional rating scales. SNPs related to BUD susceptibility were selected and obtained from the HapMap database for comparison. We aimed to clarify the mechanism underlying BQ addiction on the basis of the findings of previous genetic association studies on oral cancers and addiction and to identify potential biomarkers that can assist with early detection of BUD through interviews and psychological assessment.

## 2. Materials and Methods

### 2.1. Participants

All participants were recruited from the cancer centers of the Department of Dentistry, and the Department of General Physician at China Medical University Hospital, Taichung, Taiwan, between January 2016 and April 2019. We enrolled 50 patients with BQ chewing habits. We collected data regarding their basic demographic characteristics and rated the clinical features related to their BQ addiction. All patients provided informed consent and participated in clinical interviews conducted by a psychiatrist, with BUD defined on the basis of *Diagnostic and Statistical Manual of Mental Disorders* [Fifth Edition] (*DSM-5*) criteria. We used the criteria for the following 11 symptoms from the *DSM-5* to diagnose BUD [2]: (1) a large amount or long history of BQ consumption, (2) unsuccessful attempts at cutting down BQ use, (3) time spent chewing, (4) craving, (5) neglect of major roles, (6) social or interpersonal problems, (7) activities being given up, (8) hazardous use, (9) continued use despite knowing about associated problems, (10) tolerance, and (11) withdrawal. The diagnosis and severity of BUD were defined on the basis of patients having ≥2 of these symptoms in the past year. BQ users with 2–3 symptoms were considered to have mild BUD, those with 4–5 symptoms were considered to have moderate BUD, and those with ≥6 symptoms were considered to have severe BUD. The enrolled patients were excluded if they (1) abused illegal substances such as heroin, amphetamine, or other illicit drugs; (2) had major psychiatric disorders such as schizophrenia, bipolar disorder, major depressive disorder, antisocial personality disorder, mental disability, or developmental disorders; (3) had organic brain lesions or insults such as cerebrovascular disease, brain tumor, or head injury; (4) had any kind of cancer or cancer-related disease; and (5) were incapable of understanding and speaking Chinese. The study psychiatrist conducted semi-structured diagnostic interviews with reference to the Mini-International Neuropsychiatric Interview [22] to screen patients with the abovementioned psychiatric disorders and addictive disorders [23,24]. Diagnoses of cancer and pre-cancer were completed at the cancer center of the study hospital. Neurological diseases or other organic brain disorders were determined on the basis of the patients’ self-reports of their lifetime medical histories. Patients who were given a diagnosis of anxiety disorder or sleep disturbance and had habits of consuming alcohol, cigarettes, caffeine, or hypnotics were not excluded; such patients were allowed to enroll in the study if their hypnotic dosage had remained consistent over the past year and if they did not meet more than six criteria for other substance use disorders in the past year.

Information regarding the initial age of BQ consumption, amount of daily BQ consumption, and frequency of BQ consumption per week were obtained for all patients with BUD. We documented their oral hygiene by using the visual analog scale (VAS) and obtained data on the numbers of broken teeth they had and number of times they brushed their teeth daily through self-reports. This study was approved by the China Medical University and Hospital Research Ethics Committee (CMUH103-REC1-059, CMUH106-REC1-016).

### 2.2. Psychometric Measures of Addiction Severity

We used previously validated *DSM-5* criteria [2] to complete definite diagnosis and grading of the addiction severity of BUD. We used the Substance Use Severity Rating Scale (SUSRS) to assess BQ and alcohol consumption and cigarette smoking habits. The SUSRS was developed on the basis of diagnostic systems and the *Diagnostic and Statistical Manual of Mental Disorders* [Fourth Edition] (*DSM-IV*) and *International Classification of Diseases, Eleventh Revision* (*ICD-11*) [23,25] and contains 21 items for measuring the addictiveness of substance use for patients. It has been widely used to assess alcohol consumption, cigarette smoking, and drug use [26,27]. In the present study, a rater assessed the participants’ use of these substances using yes-or-no questions, with “yes” being provided a rating of 1 and “no” being provided a rating of 0. We used the Yale–Brown Obsessive Compulsive Disorder Rating Scale for betel quid (Y-BOCS-BQ) to define the craving severity for BQ. The Y-BOCS-BQ is designed to measure the behavioral problems of patients with BQ [28,29] and is commonly used to analyze the craving severity of substance abuse [30,31].

### 2.3. Emotional Assessment of BUD According to Severity

We used the Beck Anxiety Inventory (BAI) [32], Beck Depression Inventory (BDI) [33], and Hamilton Depression Rating Scale (HDRS) [34] to assess the emotional condition of all patients with BUD. The BAI and BDI are self-reported rating scales. Rating for the HDRS is completed by a psychiatrist.

### 2.4. Selection of Tag SNPs of Candidate Genes for Patients with BUD

We selected susceptibility genes that have been reported to be associated with candidate genes, carcinogenetic etiologies, and frequency of BQ use. These candidate genes included *MAOA* and those being associated with BQ-related carcinogenesis in oral squamous cell carcinoma (i.e., *BRCA1*, *COL9A1*, *HSPA13*, *NOTCH 1*, and *FAT1*). On the basis of the linkage disequilibrium patterns of Han Chinese people, seven tag SNPs among six genes with a minor allele frequency greater than 5% were selected from the HapMap database.

### 2.5. DNA Extraction and Genotyping

Genomic DNA was extracted from peripheral blood samples by using the Puregene DNA Isolation Kit (Gentra Systems, Minneapolis, MN, USA) in accordance with the manufacturer’s instructions. Genotyping of the 7 SNPs was performed using the Sequenom MassARRAY System by the Academia Sinica National Genotyping Center (Taipei, Taiwan). We obtained information regarding each SNP site for every participant by analyzing the results of matrix-assisted laser desorption/ionization–time-of-flight mass spectrometry with the TYPER 4.0 software (Informer Technologies, Los Angeles, CA, USA).

### 2.6. Statistical Analysis

Statistical analysis was performed using SAS 9.4 software (Cary, NC, USA). All genotype frequencies of the control population were tested for Hardy–Weinberg equilibrium. The difference between the observed and expected numbers of each genotype was compared using the chi-square test. Hardy–Weinberg equilibrium was assumed for *p* values of >0.05. The Pearson chi-square test or Fisher’s exact test was used to determine the difference in BUD and SNP genotype frequencies. Analysis of variance (ANOVA) and nonparametric ANOVA were applied to compare the demographic information of the patients with BUD and the conditions of BQ use. Because no healthy controls were included in the study, we combined the mild BUD and moderate BUD groups into a nonsevere BUD group, with the group serving as a statistical control. A logistic regression analysis model was used for emotional assessment and to investigate the association between SNPs and the severity of addictive BUD on the basis of the data of the patients with severe BUD and with nonsevere BUD.

## 3. Results

### 3.1. Basic Demographic Information of Patients with BUD According to Clinical Severity of Addiction

As presented in Table 1, the average ages of the patients with mild BUD, moderate BUD, and severe BUD were 39 (standard deviation [SD]: 13) years, 44 (SD: 7) years, and 40 (SD: 8) years, respectively. Regarding their oral hygiene, for the mild, moderate, and severe groups, the average VAS scores were 1.67 (SD: 0.78), 1.89 (SD: 0.60), and 2.10 (SD: 0.72), respectively, and the average number of tooth brushings daily were 2.75 (SD: 0.51), 2.11 (SD: 0.44), and 2.14 (SD: 0.48), respectively. The SUSRS scores for the mild, moderate, and severe groups were 2.50 (SD: 0.52), 4.44 (SD: 0.53), and 7.24 (SD: 1.02), respectively, for BQ consumption; 2.92 (SD: 3.40), 3.58 (SD: 4.06), and 3.13 (SD: 3.68), respectively, for alcohol consumption; and 2.55 (SD: 2.62), 3.00 (SD: 2.92), and 2.24 (SD: 1.48), respectively, for tobacco consumption. Only the SUSRS score for BQ consumption reached statistical significance.

### 3.2. Condition of BQ Use and Craving According to Psychometric Measures among Different Clinical Severities of BUD

As indicated in Table 2, the average initial age of BQ consumption was 20 (SD: 4) years, 17 (SD: 6) years, and 19 (SD: 5) years for the patients with mild, moderate, and severe BUD, respectively, and the average frequencies of BQ use were 3.7 (SD: 2.6) days per week, 4.8 (SD: 2.7) days per week, and 6.3 (SD: 1.7) days per week, respectively. In the mild, moderate, and severe groups, the average amount of BQ use was 12.4 (SD: 7.7) quids per day, 55.3 (SD: 63.9) quids per day, and 48.2 (SD: 45.7) quids per day, respectively, and 58.2 (SD: 61.4) quids per week, 349.7 (SD: 466.0) quids per week, and 328.2 (SD: 325.2) quids per week, respectively. Furthermore, the mild, moderate, and severe groups’ Y-BOCS-BQ were 16.83 (SD: 5.59), 23.56 (SD: 7.26), and 28.62 (SD: 7.79), respectively. With the exception of the initial age of BQ consumption, the *F* test indicated that all parameters were significant.

### 3.3. Emotional Assessment According to Different Clinical Severities of BUD

The BAI scores for emotional condition were 6.92 (SD: 6.79), 10.44 (SD: 7.60), and 17.66 (SD: 15.90) for the patients with mild, moderate, and severe BUD, respectively; their BDI and HDRS were 9.83 (SD: 11.00) and 3.42 (SD: 6.02), 16.89 (SD: 14.77) and 7.78 (SD: 5.29), and 17.79 (SD: 14.03) and 6.55 (SD: 5.05), respectively. The BAI scores were identified as significant (Table 3). As presented in Table 4, a logistic regression analysis model was used for emotional assessment of the patients with severe BUD and those with nonsevere BUD, and the odds ratio (OR) for the BAI score was 14.12 (95% confidence interval [CI], 1.66–119.98); for BDI, the score was 1.26 (95% CI, 0.35–4.49), and that for the HDRS was 2.30 (95% CI, 0.73–7.27). The findings indicated that the BAI scores were significant (Table 4). Because cravings are often associated with emotion, we also determined the OR for the Y-BOCS-BQ for craving, and its OR was significant, at 6.40 (95% CI, 1.75–23.35).

### 3.4. Association between Severity of the Addictive Property of BUD and Selected SNPs

As shown in Table 5, the results of the genome-wide association study for each allele of SNPs *BRCA1* rs2070833, *COL9A1* rs550675, *NOTCH1* rs139994842, *HSPA13* rs2822641, *FAT1* rs28647489, and *MAOA* rs2283725/rs5953210 were separated into homozygous versus heterozygous segments. The different distributions of alleles were calculated and compared between the two groups of patients with BUD, that is, the nonsevere and severe groups, with the groups defined on the basis of patients meeting fewer than or more than six *DSM-5* criteria. *MAOA* rs5953210 had an OR of 6.43 (95% CI, 5.12–7.74). Fisher’s exact test revealed no significant ORs for the other SNPs between the two groups.

## 4. Discussion

Our pilot study revealed that *DSM-5* criteria, the SUSRS, and the Y-BOCS-BQ were consistent with the addictive severity of BUD. Our analysis of the patients’ basic characteristics revealed that the initial age of BQ consumption being lower and the average amount and frequency of BQ use were associated with the clinical severity of BUD and psychometric parameters. With regard to emotional parameters, the BAI score had an OR of 14.12 (95% CI, 1.66–119.98), and craving, rated using the Y-BOCS-BQ, had an OR of 6.40 (95% CI, 1.75–23.35). Our results indicated that patients with BUD with higher addictive severity were more anxious; in addition, they had a stronger craving or desire to consume BQ. In addition, we discovered that the alleles of SNPs *BRCA1* rs2070833 and *MAOA* rs5953210 may be associated with the severity of BQ addiction. Because of our limited sample size, we performed Fischer’s exact tests and found that only the *MAOA* rs5953210 GG SNP was significant; the other SNPs related to BUD and cancers were not correlated with addiction. The abovementioned data indicated that patients with severe BUD begin to use BQ earlier, have a higher amount and frequency of BQ use, and have more self-reported anxiety symptoms on the BAI. Our findings are consistent with those of previous literature reviews indicating that BQ use resulted in more anxiety symptoms and signs. The arecoline in BQ is the main active carcinogen in the substance, and it has been ranked a first-class carcinogen [35,36]. Furthermore, the main ingredient of BQ arecoline is metabolized by MAOA. MAO enzymes are responsible for oxidative deamination of monoamine substrates, including neurotransmitters such as serotonin, histamine, dopamine, norepinephrine, and epinephrine [37,38]; these metabolized neurotransmitters influence mood [39], motivation [40], and behavior [41,42]. MAO can be classified into types A and B [43], and MAOA mainly catalyzes serotonin and MAOB, β-phenethylamine, and benzylamine [44]. Both of these isoforms enhance the oxidation of dopamine, norepinephrine, epinephrine, tyramine, and tryptamine [45]. With the exception of its role in neuropsychiatric symptoms and signs, no study has investigated the *MAOA* rs5953210 SNP as a genetic risk factor for the development of oral and pharyngeal cancers among individuals using BQ [46]. The mechanism underlying the association between the carcinogenic effects and addictive property of BQ might involve the *MAOA* rs5953210 SNP. Currently, no biomarkers or other biological examinations are available to describe the addiction severity of BUD. Most studies on BQ have investigated the carcinogenic effects of BQ use and the risk of oropharyngeal cancers among individuals who heavily chew BQ. However, overlapping expression of the *BRCA1* and *MAOA* SNPs might associate with the carcinogenic effects, heavy BQ use, and the possible addictiveness of arecoline in BQ. Several studies have focused on psychological interventions and behavioral programs to help patients with BUD stop BQ use [47,48]. Furthermore, two studies have highlighted the importance of public health and the need for government policies focused on reducing the harm from BQ [49,50]. Our pilot study provides convincing evidence regarding the biological mechanism underlying BQ addiction. A deeper understanding of the interaction between risk SNPs and pharmacotherapies for BUD would warrant the development of advanced tools for individualized prediction of addiction risk.

## 5. Conclusions

Our pilot study revealed that BQ use is associated with a risk of addiction, and the main symptoms of such addiction may be craving and anxiety. In addition, the *MAOA* rs5953210 GG SNP was noted in patients with severe BUD. Future studies should investigate the expression of different alleles in *MAOA* genotypes to gain a deeper understanding of the addictive nature of BQ. Our preliminary results potentially link the common genetic foundation of heavy BQ use, its addictive properties, and future cancer risk. Given our small sample size, studies with larger samples should be conducted to help establish potential biomarkers for accurate diagnosis of BUD. Studies on the mechanisms underlying BQ addiction could also be conducted to help reduce BQ use. Assessments of patients with different genotypes would also elucidate patients’ having different responses to certain therapeutic drugs or strategies.

## Figures and Tables

**Table 1:** Basic demographic information of patients with betel quid use disorder (BUD) with different levels of clinical severity (N = 50).

Variables	Mild BUD (N = 12)	Moderate BUD (N = 9)	Severe BUD (N = 23)	F-Test	*p*-Value	*p*-Value ***
Age (years)	39 ± 13	44 ± 7	40 ± 8	0.73	0.49	0.24
Oral Hygiene	1.67 ± 0.78	1.89 ± 0.60	2.10 ± 0.72	1.62	0.21	0.30
Teeth Brushing	2.75 ± 0.51	2.11 ± 0.44	2.14 ± 0.48	1.76	0.18	0.65
SUSRS-BQ	2.50 ± 0.52	4.44 ± 0.53	7.24 ± 1.02	139.82	<0.01 **	<0.01 **
SUSRS-Alcohol	2.92 ± 3.40	3.58 ± 4.06	3.13 ± 3.68	1.29	0.28	0.10
SUSRS-Tobacco	2.55 ± 2.62	3.00 ± 2.92	2.24 ± 1.48	1.19	0.31	0.39

Abbreviations: BQ: betel quid; SUSRS: Substance Use Severity Rating Scale; ** *p* < 0.01, statistical significance between groups. *** Nonparametric *p* value.

**Table 2:** Conditions of the use of betel quid (BQ) among patients with BQ use disorder (BUD) at different levels of clinical severity (N = 50).

Variables	Mild BUD (N = 12)	Moderate BUD (N = 9)	Severe BUD (N = 23)	F-Test	*p*-Value	*p*-Value ***
Initial consuming age (years)	20.0 ± 4.0	17.0 ± 6.0	19.0 ± 5.0	0.82	0.45	0.55
Frequency (days/per week)	3.7 ± 2.6	4.8 ± 2.7	6.3 ± 1.7	7.10	<0.01 **	<0.01 **
Amount (BQ/per day)	12.4 ± 7.7	55.3 ± 63.9	48.2 ± 45.7	3.36	0.04 *	<0.01 **
Amount (BQ/per week)	58.2 ± 61.4	349.7 ± 466.0	328.2 ± 325.2	3.44	0.04 *	<0.01 **
Y-BOCS-BQ	16.8 ± 5.6	23.6 ± 7.3	28.6 ± 7.8	11.43	<0.01 **	<0.01 **

Abbreviations: Y-BOCS-BQ: Yale–Brown Obsessive Compulsive Disorder Rating Scale for betel quid; * *p* < 0.05 and ** *p* < 0.01, statistical significance between groups. *** Nonparametric *p* value.

**Table 3:** Association between emotional condition and addiction severity in patients with betel quid use disorder (BUD).

Variables	Mild BUD (N = 12)	Moderate BUD (N = 9)	Severe BUD (N = 29)	F-Test	*p*-Value
BAI	6.92 ± 6.79	10.44 ± 7.60	17.66 ± 15.90	3.22	<0.05 *
BDI	9.83 ± 11.00	16.89 ± 14.77	17.79 ± 14.03	1.51	0.23
HDRS	3.42 ± 6.02	7.78 ± 5.29	6.55 ± 5.05	2.06	0.14

Abbreviations: BAI: Beck Anxiety Inventory; BDI: Beck Depression Inventory; HDRS: Hamilton Depression Rating Scale; * *p* < 0.05, statistical significance between groups.

**Table 4:** Association between severity of betel quid (BQ) addiction and emotional condition of patients with BQ use disorder (BUD) (N = 50).

Variables	OR	95% CI	*p*-Value
BAI	14.12	1.66–119.98	0.02 *
BDI	1.26	0.35–4.49	0.73
HDRS	2.30	0.73–7.27	0.16
Y-BOCS-BQ	6.40	1.75–23.35	<0.01 **

Abbreviations: BAI: Beck Anxiety Inventory; BDI: Beck Depression Inventory; CI: confidence interval; HDRS: Hamilton Depression Rating Scale; OR: odds ratio; Y-BOCS-BQ: Yale–Brown Obsessive Compulsive Disorder Rating Scale for betel quid; * *p* < 0.05 and ** *p* < 0.01, statistical significance between groups.

**Table 5:** Association between severity level of addictive betel quid use disorder (BUD) and single nucleotide polymorphisms (SNPs) in selected alleles (N = 50).

Gene	SNPs Genotypes	Severe BUD (N = 34)	Non-Severe BUD (N = 16)	OR (95% CI)	χ2 Test	Fischer’s Test
*BRCA1*	rs2070833					
	CC (AA)	28	8	1		
	CA	6	8	4.67 (3.34–5.98)	0.02 *	0.11
*COL9A1*	rs550675					
	CC (TT)	23	8	1		
	CT	11	8	2.09 (0.88–3.31)	0.230	0.18
*NOTCH 1*	rs139994842					
	GG	32	15	1		
	GA	2	1	1.07 (−1.41–3.54)	0.959	0.69
*HSPA13*	rs2822641					
	GG	29	15	1		
	GT	5	1	0.39 (−1.85–2.62)	0.391	0.92
*FAT1*	rs28647489					
	GG (AA)	17	9	1		
	AG	17	7	0.78 (−0.42–1.97)	0.680	0.63
*MAOA*	rs2283725					
	CC	22	10	1		
	TT	12	6	1.1 (−0.13–2.33)	0.880	0.52
*MAOA*	rs5953210					
	GG	27	6	1		
	AA	7	10	6.43 (5.12–7.74)	<0.01 **	<0.01 **

Abbreviations: CI: confidence interval; OR: odds ratio; *p* value calculated using the chi-square test and Fischer’s exact test, * *p* < 0.05 and ** *p* < 0.01, statistical significance between groups.

## Data Availability

Data are contained within the article.
